# 2-(3-Methyl-2-oxoquinoxalin-1-yl)-*N*-(4-methyl­phen­yl)acetamide

**DOI:** 10.1107/S2414314623003577

**Published:** 2023-04-21

**Authors:** Mohcine Missioui, Abdulsalam Alsubari, Joel T. Mague, El Mokhtar Essassi, Youssef Ramli

**Affiliations:** a Laboratory of Medicinal Chemistry, Drug Sciences Research Center, Faculty of Medicine and Pharmacy, Mohammed V University in Rabat, Morocco; b Laboratory of Medicinal Chemistry, Faculty of Clinical Pharmacy, 21 September University, Yemen; c Department of Chemistry, Tulane University, New Orleans, LA 70118, USA; d Laboratory of Heterocyclic Organic Chemistry, Faculty of Sciences, Mohammed V University, Rabat, Morocco; e Mohammed VI Center for Research and Innovation (CM6), Rabat 10000, Morocco; Katholieke Universiteit Leuven, Belgium

**Keywords:** crystal structure, hydro­gen bond, edge-to-edge π-inter­action, amide, quinoxaline

## Abstract

The quinoxaline moiety in the title mol­ecule, C_18_H_17_N_3_O_2_, is not quite planar and the *p*-tolyl group is rotationally disordered over two nearly equally populated sets of sites. In the crystal, N—H⋯O and C—H⋯O hydro­gen bonds form chains extending along the *b*-axis direction. Due to the disorder of the *p*-tolyl rings, short C⋯C distances are observed between adjacent chains.

## Abstract 2

Chains extending along the *b*-axis direction are formed through hydro­gen bonding between the amide functions of the title quinoxaline derivative.

## Graphical abstract

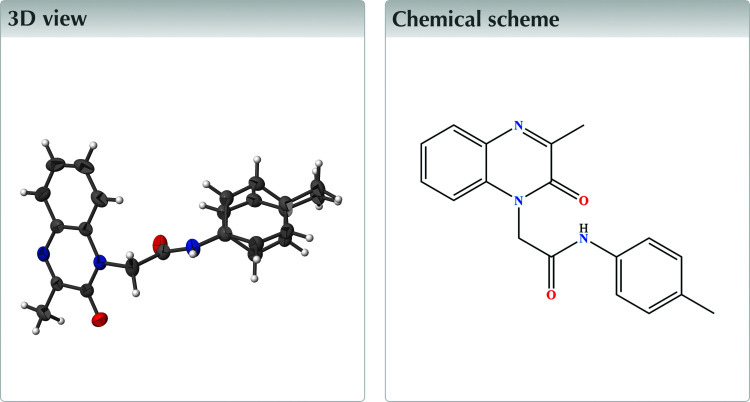

## Structure description

**Figure 1:**
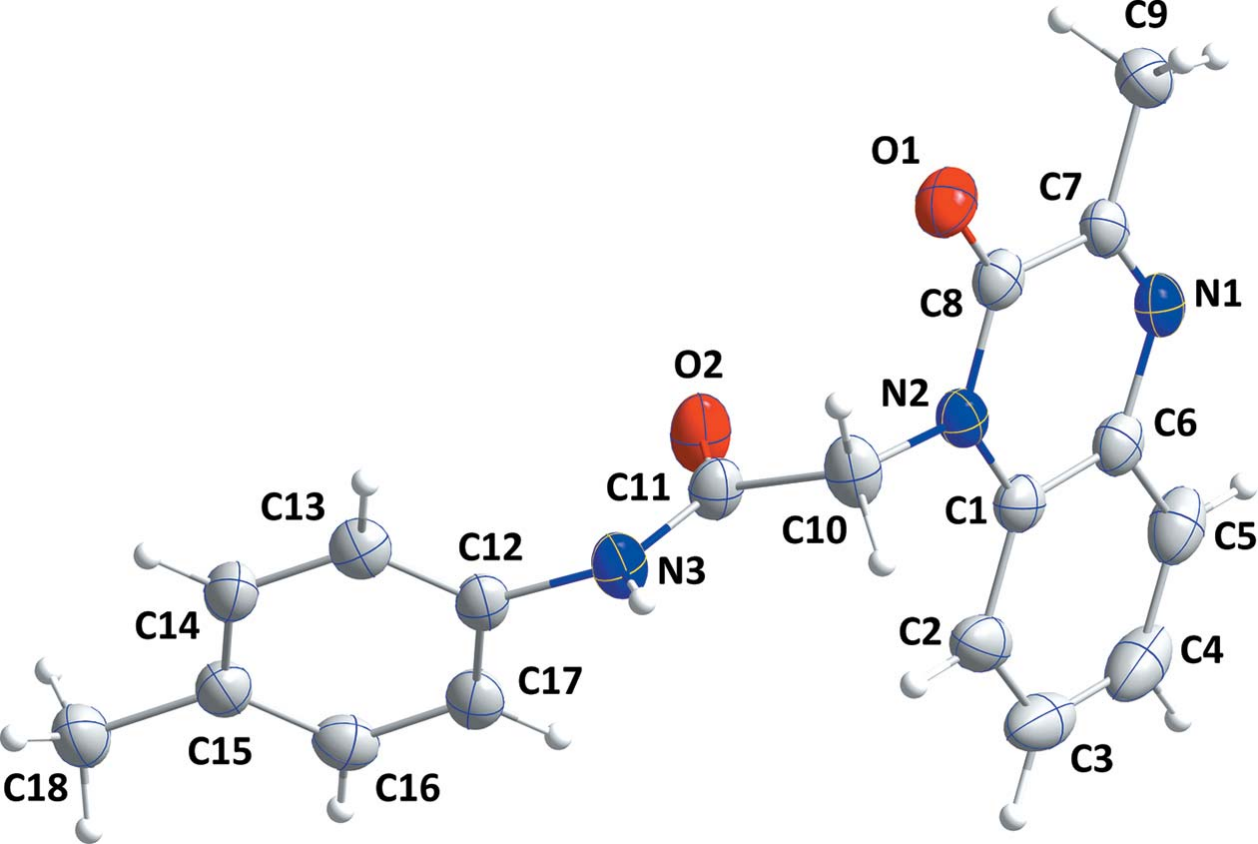
The title mol­ecule with the atom-labelling scheme and 50% probability displacement ellipsoids. Only the major orientation of the disordered *p*-tolyl group is shown.

Among the various classes of nitro­gen heterocyclic compounds, quinoxaline derivatives display a broad spectrum of biological and pharmacological activities (Ramli & Essassi, 2015 ▸). Some analogs have been synthesized and evaluated for their industrial properties (*e.g.* Lgaz *et al.*, 2015 ▸). As a continuation of our work in this area (*e.g.* Abad *et al.*, 2021 ▸), the title compound was synthesized and its crystal structure is reported here (Fig. 1 ▸).

**Figure 2:**
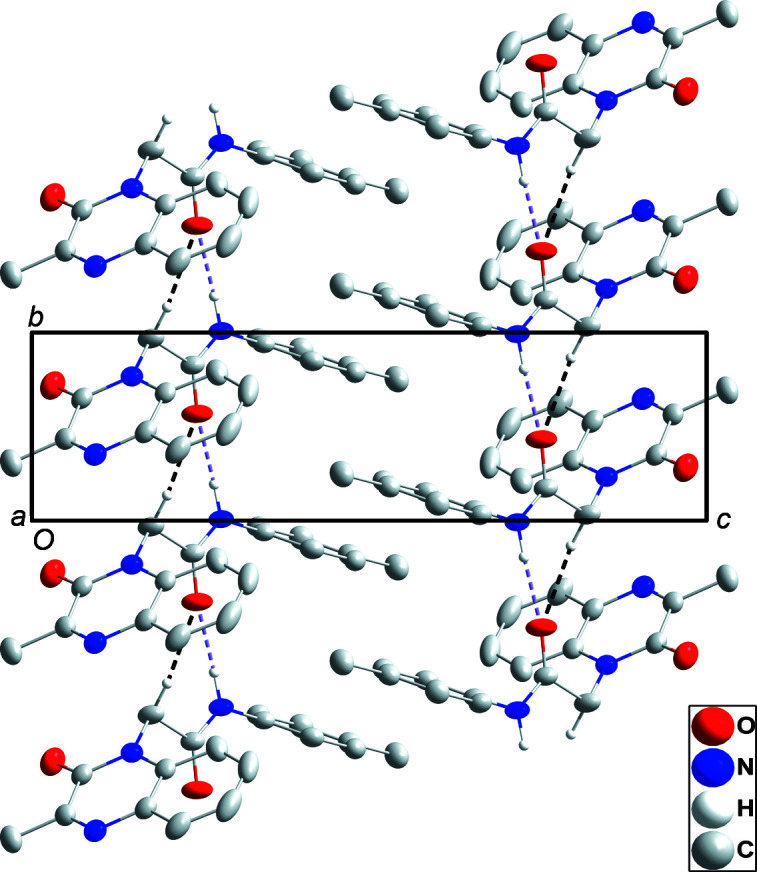
A portion of one ribbon, viewed along the *a*-axis direction, with N—H⋯O and C—H⋯O hydro­gen bonds depicted, respectively, by violet and black dashed lines. Non-inter­acting H atoms have been omitted for clarity.

The quinoxaline moiety is slightly nonplanar as there is a dihedral angle of 1.26 (14)° between the mean planes through the constituent rings. The *p*-tolyl ring is rotationally disordered over two orientations 45.46 (18)° apart in a 0.503 (2):0.497 (2) ratio. In the crystal, N3—H3*A*⋯O2 and C10—H10*B*⋯O2 hydro­gen bonds (Table 1 ▸) form chains of mol­ecules extending along the *b*-axis direction. Pairs of inversion-related chains show C16⋯C17^i^ and C17⋯C16^i^ [symmetry code: (i) −*x* + 1, −*y*, −*z* + 1] distances of 2.695 (4) Å, which is 0.71 Å less than the sum of the van der Waals radii and is likely due to the disorder involving this ring. The chains stack along the *c*-axis direction (Figs. 2 ▸ and 3 ▸).

## Synthesis and crystallization

1.00 g (6.24 mmol) of 3-methyl­quinoxalin-2(1*H*)-one was dissolved in 25 ml of di­methyl­formamide and 1.15 g (6.24 mmol) of 2-chloro-*N*-(*p*-tol­yl)acetamide were added, followed by 1.0 g (7.5 mmol) of potassium bicarbonate, and a spatula tip of BTBA (benzyl­tri­butyl­ammonium chloride) was used as a phase-transfer catalyst. The reaction was stirred for 2 h under reflux at 353 K. When the starting reagents had completely reacted, 500 ml of distilled water were added and a few minutes later the product precipitated. This was filtered off, dried and recrystallized from hot ethanol solution to yield light-yellow plate-like crystals of the title compound.

## Refinement

Crystal, data collection and refinement details are presented in Table 2 ▸. H atoms attached to carbon were included as riding atoms in idealized positions with isotropic displacement parameters tied to those of the attached atoms, while that attached to nitro­gen was refined independently. Analysis of 446 reflections having *I*/σ(*I*) > 20 and chosen from the full data set with *CELL_NOW* (Sheldrick, 2008 ▸) showed the crystal to belong to the monoclinic system and to be twinned by a 180° rotation about the c* axis. The structure was refined as a two-component twin. The two components [0.503 (2):0.497 (2) ratio] of the disordered C12–C17 ring were refined as rigid hexa­gons.

## Supplementary Material

Crystal structure: contains datablock(s) global, I. DOI: 10.1107/S2414314623003577/vm4060sup1.cif

Structure factors: contains datablock(s) I. DOI: 10.1107/S2414314623003577/vm4060Isup2.hkl

Supporting information file. DOI: 10.1107/S2414314623003577/vm4060Isup3.cml

CCDC reference: 2254194

Additional supporting information:  crystallographic information; 3D view; checkCIF report

## Figures and Tables

**Figure 3:**
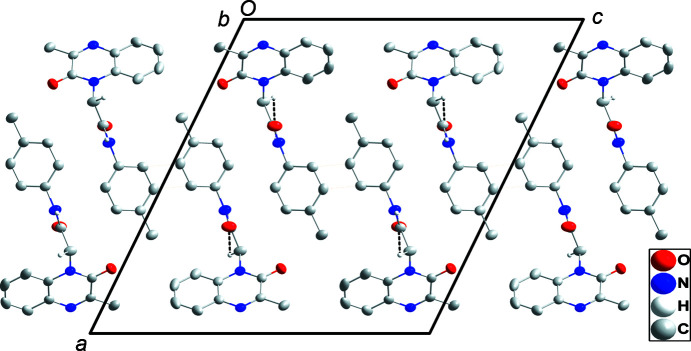
The packing, viewed along the *b*-axis direction, with inter­molecular inter­actions depicted as in Fig. 2 ▸ and non-inter­acting H atoms omitted for clarity.

**Table 1:** Hydrogen-bond geometry (Å, °)

*D*—H⋯*A*	*D*—H	H⋯*A*	*D*⋯*A*	*D*—H⋯*A*
N3—H3*A*⋯O2^i^	0.89 (3)	1.91 (3)	2.790 (2)	167 (3)
C10—H10*B*⋯O2^i^	0.99	2.57	3.179 (3)	120

Symmetry code: (i) 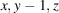 .

**Table 2:** Experimental details

Crystal data	
Chemical formula	C_18_H_17_N_3_O_2_
*M*_r_	307.34
Crystal system, space group	Monoclinic, *P*2_1_/*c*
Temperature (K)	125
*a*, *b*, *c* (Å)	19.2935 (7), 4.6933 (2), 18.7498 (7)
β (°)	116.106 (2)
*V* (Å^3^)	1524.59 (11)
*Z*	4
Radiation type	Cu *K*α
μ (mm^−1^)	0.72
Crystal size (mm)	0.23 × 0.15 × 0.02

Data collection	
Diffractometer	Bruker D8 VENTURE PHOTON 3 CPAD
Absorption correction	Multi-scan (*TWINABS*; Sheldrick, 2009 ▸)
*T*_min_, *T*_max_	0.85, 0.99
No. of measured, independent and observed [*I* > 2σ(*I*)] reflections	5971, 5971, 4844
*R*_int_	0.065
(sin θ/λ)_max_ (Å^−1^)	0.619

Refinement	
*R*[*F* ^2^ > 2σ(*F* ^2^)], *wR*(*F* ^2^), *S*	0.057, 0.158, 1.04
No. of reflections	5971
No. of parameters	214
No. of restraints	4
H-atom treatment	H atoms treated by a mixture of independent and constrained refinement
Δρ_max_, Δρ_min_ (e Å^−3^)	0.30, −0.32

Computer programs: *APEX4* (Bruker, 2021 ▸), *SAINT* (Bruker, 2021 ▸), *SHELXT* (Sheldrick, 2015*a* ▸), *SHELXL2018* (Sheldrick, 2015*b* ▸), *DIAMOND* (Brandenburg & Putz, 2012 ▸) and *SHELXTL* (Bruker, 2021 ▸).

## full crystallographic data

### Crystal data

**Table:**

C_18_H_17_N_3_O_2_	*F*(000) = 648
*M**_r_* = 307.34	*D*_x_ = 1.339 Mg m^−^^3^
Monoclinic, *P*2_1_/*c*	Cu *K*α radiation, λ = 1.54178 Å
*a* = 19.2935 (7) Å	Cell parameters from 9934 reflections
*b* = 4.6933 (2) Å	θ = 4.7–72.5°
*c* = 18.7498 (7) Å	µ = 0.72 mm^−^^1^
β = 116.106 (2)°	*T* = 125 K
*V* = 1524.59 (11) Å^3^	Plate, colourless
*Z* = 4	0.23 × 0.15 × 0.02 mm

### Data collection

**Table:**

Bruker D8 VENTURE PHOTON 3 CPAD diffractometer	5971 independent reflections
Radiation source: INCOATEC IµS micro–focus source	4844 reflections with *I* > 2σ(*I*)
Mirror monochromator	*R*_int_ = 0.065
Detector resolution: 7.3910 pixels mm^-1^	θ_max_ = 72.6°, θ_min_ = 2.6°
φ and ω scans	*h* = −23→21
Absorption correction: multi-scan (*TWINABS*; Sheldrick, 2009)	*k* = 0→5
*T*_min_ = 0.85, *T*_max_ = 0.99	*l* = 0→23
5971 measured reflections	

### Refinement

**Table:**

Refinement on *F*^2^	Primary atom site location: dual
Least-squares matrix: full	Secondary atom site location: difference Fourier map
*R*[*F*^2^ > 2σ(*F*^2^)] = 0.057	Hydrogen site location: mixed
*wR*(*F*^2^) = 0.158	H atoms treated by a mixture of independent and constrained refinement
*S* = 1.04	*w* = 1/[σ^2^(*F*_o_^2^) + (0.0668*P*)^2^ + 0.6811*P*] where *P* = (*F*_o_^2^ + 2*F*_c_^2^)/3
5971 reflections	(Δ/σ)_max_ < 0.001
214 parameters	Δρ_max_ = 0.30 e Å^−^^3^
4 restraints	Δρ_min_ = −0.32 e Å^−^^3^

### Special details

**Table:**

Experimental. The diffraction data were obtained from 16 sets of frames, each of width 0.5° in ω or φ, collected with scan parameters determined by the "strategy" routine in *APEX4*. The scan time was θ-dependent and ranged from 5 to 10 sec/frame. Analysis of 446 reflections having I/σ(I) > 20 and chosen from the full data set with *CELL_NOW* (Sheldrick, 2008) showed the crystal to belong to the monoclinic system and to be twinned by a 180° rotation about the *c** axis. The raw data were processed using the multi-component version of *SAINT* under control of the two-component orientation file generated by *CELL_NOW*.
Geometry. All esds (except the esd in the dihedral angle between two l.s. planes) are estimated using the full covariance matrix. The cell esds are taken into account individually in the estimation of esds in distances, angles and torsion angles; correlations between esds in cell parameters are only used when they are defined by crystal symmetry. An approximate (isotropic) treatment of cell esds is used for estimating esds involving l.s. planes.
Refinement. Refinement of F^2^ against ALL reflections. The weighted R-factor wR and goodness of fit S are based on F^2^, conventional R-factors R are based on F, with F set to zero for negative F^2^. The threshold expression of F^2^ > 2sigma(F^2^) is used only for calculating R-factors(gt) etc. and is not relevant to the choice of reflections for refinement. R-factors based on F^2^ are statistically about twice as large as those based on F, and R- factors based on ALL data will be even larger. H-atoms attached to carbon were placed in calculated positions (C—H = 0.95 - 0.99 Å) and were included as riding contributions with isotropic displacement parameters 1.2 - 1.5 times those of the attached atoms. That attached to nitrogen was refined independently. Refined as a 2-component twin. The C12···C17 ring is rotationally disordered over two sites in a 0.503 (2)/0.497 (2) ratio. The two rings were refined as rigid hexagons.

### Fractional atomic coordinates and isotropic or equivalent isotropic displacement parameters (Å^2^)

**Table:**

	*x*	*y*	*z*	*U*_iso_*/*U*_eq_	Occ. (<1)
O1	0.79390 (9)	0.2813 (4)	0.96921 (10)	0.0428 (4)	
O2	0.66001 (9)	0.4321 (3)	0.75510 (11)	0.0439 (4)	
N1	0.91798 (10)	0.6484 (4)	0.90616 (11)	0.0328 (4)	
N2	0.80138 (10)	0.2332 (4)	0.85180 (10)	0.0303 (4)	
N3	0.60746 (11)	−0.0081 (4)	0.71946 (12)	0.0346 (4)	
H3A	0.6168 (16)	−0.194 (7)	0.7296 (16)	0.048 (8)*	
C1	0.83439 (12)	0.3204 (5)	0.80215 (12)	0.0303 (5)	
C2	0.81095 (14)	0.2121 (6)	0.72561 (14)	0.0425 (6)	
H2	0.771477	0.071925	0.705499	0.051*	
C3	0.84517 (17)	0.3090 (7)	0.67925 (15)	0.0522 (8)	
H3	0.828981	0.235039	0.627189	0.063*	
C4	0.90311 (16)	0.5136 (7)	0.70797 (16)	0.0527 (8)	
H4	0.926420	0.578486	0.675630	0.063*	
C5	0.92664 (14)	0.6221 (6)	0.78309 (15)	0.0433 (6)	
H5	0.966159	0.762259	0.802523	0.052*	
C6	0.89276 (12)	0.5272 (5)	0.83112 (12)	0.0322 (5)	
C7	0.88615 (11)	0.5649 (5)	0.95044 (12)	0.0305 (5)	
C8	0.82374 (12)	0.3501 (5)	0.92579 (12)	0.0306 (5)	
C9	0.91077 (14)	0.6936 (6)	1.03080 (14)	0.0426 (6)	
H9A	0.952575	0.830084	1.041179	0.064*	
H9B	0.929002	0.543057	1.071109	0.064*	
H9C	0.866893	0.792162	1.032824	0.064*	
C10	0.73747 (12)	0.0317 (5)	0.82339 (15)	0.0377 (5)	
H10A	0.728584	−0.036647	0.868641	0.045*	
H10B	0.750558	−0.134921	0.799270	0.045*	
C11	0.66396 (12)	0.1729 (4)	0.76175 (13)	0.0316 (5)	
C12	0.53264 (11)	0.0608 (7)	0.65663 (13)	0.0294 (5)	0.503 (2)
C13	0.46472 (15)	0.0306 (7)	0.66460 (13)	0.0336 (7)	0.503 (2)
H13	0.466547	−0.031354	0.713638	0.040*	0.503 (2)
C14	0.39412 (11)	0.0910 (7)	0.60083 (17)	0.0356 (9)	0.503 (2)
H14	0.347698	0.070370	0.606282	0.043*	0.503 (2)
C15	0.39144 (11)	0.1817 (7)	0.52909 (14)	0.0343 (7)	0.503 (2)
C16	0.45936 (15)	0.2119 (7)	0.52111 (12)	0.0354 (7)	0.503 (2)
H16	0.457528	0.273880	0.472078	0.042*	0.503 (2)
C17	0.52996 (12)	0.1515 (7)	0.58488 (16)	0.0334 (7)	0.503 (2)
H17	0.576378	0.172158	0.579433	0.040*	0.503 (2)
C18	0.3136 (3)	0.2476 (16)	0.4594 (4)	0.0433 (14)	0.503 (2)
H18A	0.317427	0.220882	0.409396	0.065*	0.503 (2)
H18B	0.299208	0.445281	0.463168	0.065*	0.503 (2)
H18C	0.274261	0.118941	0.460865	0.065*	0.503 (2)
C12A	0.53474 (11)	0.0803 (6)	0.65812 (15)	0.0294 (5)	0.497 (2)
C13A	0.47318 (16)	−0.1031 (5)	0.64255 (17)	0.0336 (7)	0.497 (2)
H13A	0.480998	−0.274459	0.672089	0.040*	0.497 (2)
C14A	0.40017 (13)	−0.0359 (6)	0.58377 (18)	0.0356 (9)	0.497 (2)
H14A	0.358098	−0.161254	0.573130	0.043*	0.497 (2)
C15A	0.38873 (11)	0.2148 (6)	0.54055 (15)	0.0343 (7)	0.497 (2)
C16A	0.45030 (16)	0.3982 (5)	0.55611 (16)	0.0354 (7)	0.497 (2)
H16A	0.442477	0.569545	0.526577	0.042*	0.497 (2)
C17A	0.52330 (13)	0.3310 (6)	0.61490 (17)	0.0334 (7)	0.497 (2)
H17A	0.565379	0.456344	0.625535	0.040*	0.497 (2)
C18A	0.3093 (3)	0.295 (2)	0.4756 (3)	0.0433 (14)	0.497 (2)
H18D	0.313503	0.340756	0.426650	0.065*	0.497 (2)
H18E	0.289595	0.461028	0.492575	0.065*	0.497 (2)
H18F	0.273790	0.134311	0.465835	0.065*	0.497 (2)

### Atomic displacement parameters (Å^2^)

**Table:**

	*U*^11^	*U*^22^	*U*^33^	*U*^12^	*U*^13^	*U*^23^
O1	0.0390 (9)	0.0533 (11)	0.0416 (9)	0.0026 (8)	0.0228 (8)	0.0094 (8)
O2	0.0358 (9)	0.0220 (8)	0.0660 (11)	0.0024 (6)	0.0151 (8)	0.0024 (7)
N1	0.0256 (9)	0.0336 (10)	0.0346 (10)	0.0029 (7)	0.0092 (7)	0.0034 (8)
N2	0.0276 (9)	0.0268 (9)	0.0331 (9)	0.0009 (7)	0.0101 (7)	0.0007 (7)
N3	0.0316 (10)	0.0211 (9)	0.0422 (11)	0.0031 (7)	0.0081 (8)	0.0018 (8)
C1	0.0280 (10)	0.0300 (11)	0.0298 (10)	0.0093 (8)	0.0099 (8)	0.0038 (8)
C2	0.0411 (13)	0.0457 (14)	0.0338 (12)	0.0143 (11)	0.0102 (10)	−0.0042 (10)
C3	0.0554 (16)	0.0694 (19)	0.0293 (12)	0.0316 (15)	0.0164 (11)	0.0065 (12)
C4	0.0492 (15)	0.074 (2)	0.0444 (14)	0.0285 (14)	0.0295 (13)	0.0257 (14)
C5	0.0354 (12)	0.0525 (15)	0.0451 (13)	0.0106 (11)	0.0206 (11)	0.0185 (12)
C6	0.0276 (10)	0.0348 (12)	0.0327 (11)	0.0087 (9)	0.0118 (9)	0.0082 (9)
C7	0.0248 (10)	0.0321 (11)	0.0293 (10)	0.0057 (8)	0.0069 (8)	0.0017 (9)
C8	0.0281 (10)	0.0315 (11)	0.0309 (11)	0.0068 (8)	0.0120 (9)	0.0069 (9)
C9	0.0352 (12)	0.0509 (15)	0.0330 (12)	0.0056 (11)	0.0070 (10)	−0.0068 (11)
C10	0.0311 (11)	0.0260 (11)	0.0474 (13)	−0.0009 (9)	0.0094 (10)	0.0041 (10)
C11	0.0298 (11)	0.0241 (11)	0.0393 (12)	0.0031 (8)	0.0137 (9)	0.0022 (9)
C12	0.0282 (10)	0.0218 (10)	0.0350 (11)	0.0016 (8)	0.0111 (9)	−0.0008 (8)
C13	0.0365 (16)	0.027 (2)	0.0366 (18)	−0.0007 (16)	0.0152 (13)	0.0021 (14)
C14	0.0287 (14)	0.039 (3)	0.040 (2)	−0.0009 (17)	0.0154 (13)	−0.0033 (17)
C15	0.0321 (12)	0.0319 (14)	0.0353 (14)	0.0028 (10)	0.0115 (10)	−0.0037 (11)
C16	0.0434 (17)	0.0296 (16)	0.0317 (16)	0.0012 (13)	0.0152 (13)	−0.0006 (12)
C17	0.0326 (15)	0.0264 (15)	0.0400 (17)	−0.0025 (13)	0.0150 (13)	−0.0024 (12)
C18	0.0352 (14)	0.046 (3)	0.039 (3)	0.0052 (14)	0.0082 (17)	−0.005 (2)
C12A	0.0282 (10)	0.0218 (10)	0.0350 (11)	0.0016 (8)	0.0111 (9)	−0.0008 (8)
C13A	0.0365 (16)	0.027 (2)	0.0366 (18)	−0.0007 (16)	0.0152 (13)	0.0021 (14)
C14A	0.0287 (14)	0.039 (3)	0.040 (2)	−0.0009 (17)	0.0154 (13)	−0.0033 (17)
C15A	0.0321 (12)	0.0319 (14)	0.0353 (14)	0.0028 (10)	0.0115 (10)	−0.0037 (11)
C16A	0.0434 (17)	0.0296 (16)	0.0317 (16)	0.0012 (13)	0.0152 (13)	−0.0006 (12)
C17A	0.0326 (15)	0.0264 (15)	0.0400 (17)	−0.0025 (13)	0.0150 (13)	−0.0024 (12)
C18A	0.0352 (14)	0.046 (3)	0.039 (3)	0.0052 (14)	0.0082 (17)	−0.005 (2)

### Geometric parameters (Å, º)

**Table:**

O1—C8	1.229 (3)	C12—C13	1.3900
O2—C11	1.222 (3)	C12—C17	1.3900
N1—C7	1.292 (3)	C13—C14	1.3900
N1—C6	1.392 (3)	C13—H13	0.9500
N2—C8	1.373 (3)	C14—C15	1.3900
N2—C1	1.401 (3)	C14—H14	0.9500
N2—C10	1.456 (3)	C15—C16	1.3900
N3—C11	1.333 (3)	C15—C18	1.527 (3)
N3—C12A	1.429 (2)	C16—C17	1.3900
N3—C12	1.442 (2)	C16—H16	0.9500
N3—H3A	0.89 (3)	C17—H17	0.9500
C1—C2	1.397 (3)	C18—H18A	0.9800
C1—C6	1.402 (3)	C18—H18B	0.9800
C2—C3	1.379 (4)	C18—H18C	0.9800
C2—H2	0.9500	C12A—C13A	1.3900
C3—C4	1.390 (4)	C12A—C17A	1.3900
C3—H3	0.9500	C13A—C14A	1.3900
C4—C5	1.373 (4)	C13A—H13A	0.9500
C4—H4	0.9500	C14A—C15A	1.3900
C5—C6	1.398 (3)	C14A—H14A	0.9500
C5—H5	0.9500	C15A—C16A	1.3900
C7—C8	1.480 (3)	C15A—C18A	1.527 (3)
C7—C9	1.493 (3)	C16A—C17A	1.3900
C9—H9A	0.9800	C16A—H16A	0.9500
C9—H9B	0.9800	C17A—H17A	0.9500
C9—H9C	0.9800	C18A—H18D	0.9800
C10—C11	1.531 (3)	C18A—H18E	0.9800
C10—H10A	0.9900	C18A—H18F	0.9800
C10—H10B	0.9900		
			
C7—N1—C6	118.2 (2)	C13—C12—C17	120.0
C8—N2—C1	121.48 (19)	C13—C12—N3	122.8 (2)
C8—N2—C10	117.92 (19)	C17—C12—N3	117.2 (2)
C1—N2—C10	120.41 (19)	C12—C13—C14	120.0
C11—N3—C12A	123.3 (2)	C12—C13—H13	120.0
C11—N3—C12	127.3 (2)	C14—C13—H13	120.0
C11—N3—H3A	117.3 (18)	C13—C14—C15	120.0
C12A—N3—H3A	119.3 (18)	C13—C14—H14	120.0
C12—N3—H3A	115.4 (18)	C15—C14—H14	120.0
C2—C1—N2	122.7 (2)	C16—C15—C14	120.0
C2—C1—C6	119.4 (2)	C16—C15—C18	120.4 (4)
N2—C1—C6	117.85 (19)	C14—C15—C18	119.6 (4)
C3—C2—C1	119.9 (3)	C15—C16—C17	120.0
C3—C2—H2	120.0	C15—C16—H16	120.0
C1—C2—H2	120.0	C17—C16—H16	120.0
C2—C3—C4	120.7 (2)	C16—C17—C12	120.0
C2—C3—H3	119.7	C16—C17—H17	120.0
C4—C3—H3	119.7	C12—C17—H17	120.0
C5—C4—C3	120.0 (2)	C15—C18—H18A	109.5
C5—C4—H4	120.0	C15—C18—H18B	109.5
C3—C4—H4	120.0	H18A—C18—H18B	109.5
C4—C5—C6	120.4 (3)	C15—C18—H18C	109.5
C4—C5—H5	119.8	H18A—C18—H18C	109.5
C6—C5—H5	119.8	H18B—C18—H18C	109.5
N1—C6—C5	117.8 (2)	C13A—C12A—C17A	120.0
N1—C6—C1	122.57 (19)	C13A—C12A—N3	115.8 (2)
C5—C6—C1	119.6 (2)	C17A—C12A—N3	124.2 (2)
N1—C7—C8	123.88 (19)	C12A—C13A—C14A	120.0
N1—C7—C9	119.7 (2)	C12A—C13A—H13A	120.0
C8—C7—C9	116.4 (2)	C14A—C13A—H13A	120.0
O1—C8—N2	122.2 (2)	C15A—C14A—C13A	120.0
O1—C8—C7	121.7 (2)	C15A—C14A—H14A	120.0
N2—C8—C7	116.01 (18)	C13A—C14A—H14A	120.0
C7—C9—H9A	109.5	C16A—C15A—C14A	120.0
C7—C9—H9B	109.5	C16A—C15A—C18A	118.7 (5)
H9A—C9—H9B	109.5	C14A—C15A—C18A	121.3 (5)
C7—C9—H9C	109.5	C15A—C16A—C17A	120.0
H9A—C9—H9C	109.5	C15A—C16A—H16A	120.0
H9B—C9—H9C	109.5	C17A—C16A—H16A	120.0
N2—C10—C11	110.41 (17)	C16A—C17A—C12A	120.0
N2—C10—H10A	109.6	C16A—C17A—H17A	120.0
C11—C10—H10A	109.6	C12A—C17A—H17A	120.0
N2—C10—H10B	109.6	C15A—C18A—H18D	109.5
C11—C10—H10B	109.6	C15A—C18A—H18E	109.5
H10A—C10—H10B	108.1	H18D—C18A—H18E	109.5
O2—C11—N3	125.1 (2)	C15A—C18A—H18F	109.5
O2—C11—C10	120.2 (2)	H18D—C18A—H18F	109.5
N3—C11—C10	114.60 (18)	H18E—C18A—H18F	109.5
			
C8—N2—C1—C2	−177.8 (2)	C12—N3—C11—O2	2.3 (4)
C10—N2—C1—C2	−2.9 (3)	C12A—N3—C11—C10	−178.9 (2)
C8—N2—C1—C6	1.5 (3)	C12—N3—C11—C10	−178.8 (2)
C10—N2—C1—C6	176.39 (18)	N2—C10—C11—O2	−15.5 (3)
N2—C1—C2—C3	179.3 (2)	N2—C10—C11—N3	165.5 (2)
C6—C1—C2—C3	0.0 (3)	C11—N3—C12—C13	−110.6 (3)
C1—C2—C3—C4	0.1 (4)	C11—N3—C12—C17	71.6 (3)
C2—C3—C4—C5	−0.2 (4)	C17—C12—C13—C14	0.0
C3—C4—C5—C6	0.2 (4)	N3—C12—C13—C14	−177.7 (3)
C7—N1—C6—C5	178.99 (19)	C12—C13—C14—C15	0.0
C7—N1—C6—C1	0.2 (3)	C13—C14—C15—C16	0.0
C4—C5—C6—N1	−178.9 (2)	C13—C14—C15—C18	−179.99 (8)
C4—C5—C6—C1	−0.1 (3)	C14—C15—C16—C17	0.0
C2—C1—C6—N1	178.75 (19)	C18—C15—C16—C17	179.99 (8)
N2—C1—C6—N1	−0.6 (3)	C15—C16—C17—C12	0.0
C2—C1—C6—C5	0.0 (3)	C13—C12—C17—C16	0.0
N2—C1—C6—C5	−179.35 (19)	N3—C12—C17—C16	177.8 (3)
C6—N1—C7—C8	−0.7 (3)	C11—N3—C12A—C13A	−154.7 (2)
C6—N1—C7—C9	−178.98 (19)	C11—N3—C12A—C17A	25.5 (4)
C1—N2—C8—O1	178.05 (19)	C17A—C12A—C13A—C14A	0.0
C10—N2—C8—O1	3.1 (3)	N3—C12A—C13A—C14A	−179.8 (3)
C1—N2—C8—C7	−2.0 (3)	C12A—C13A—C14A—C15A	0.0
C10—N2—C8—C7	−176.93 (17)	C13A—C14A—C15A—C16A	0.0
N1—C7—C8—O1	−178.4 (2)	C13A—C14A—C15A—C18A	179.99 (8)
C9—C7—C8—O1	−0.1 (3)	C14A—C15A—C16A—C17A	0.0
N1—C7—C8—N2	1.6 (3)	C18A—C15A—C16A—C17A	−179.99 (7)
C9—C7—C8—N2	179.90 (18)	C15A—C16A—C17A—C12A	0.0
C8—N2—C10—C11	104.1 (2)	C13A—C12A—C17A—C16A	0.0
C1—N2—C10—C11	−71.0 (2)	N3—C12A—C17A—C16A	179.8 (3)
C12A—N3—C11—O2	2.2 (4)		

### Hydrogen-bond geometry (Å, º)

**Table:**

*D*—H···*A*	*D*—H	H···*A*	*D*···*A*	*D*—H···*A*
N3—H3*A*···O2^i^	0.89 (3)	1.91 (3)	2.790 (2)	167 (3)
C10—H10*B*···O2^i^	0.99	2.57	3.179 (3)	120

Symmetry code: (i) *x*, *y*−1, *z*.
